# A rare SNP mutation in *Brachytic2* moderately reduces plant height and increases yield potential in maize

**DOI:** 10.1093/jxb/erv182

**Published:** 2015-04-28

**Authors:** Anqi Xing, Yufeng Gao, Lingfeng Ye, Weiping Zhang, Lichun Cai, Ada Ching, Victor Llaca, Blaine Johnson, Lin Liu, Xiaohong Yang, Dingming Kang, Jianbing Yan, Jiansheng Li

**Affiliations:** ^1^National Maize Improvement Centre of China, China Agricultural University, Beijing 100193, China; ^2^College of Agronomy and Biotechnology, China Agricultural University, Beijing 100193, China; ^3^DuPont Co., Agricultural Biotechnology, 200 Powder Mill Road, Wilmington, DE 19805, USA; ^4^Pioneer Hi-Bred Intl, 1501 Road P, York, NE 68467-8234, USA; ^5^National Key Laboratory of Crop Genetic Improvement, Huazhong Agricultural University, Wuhan 430070, China

**Keywords:** *Brachytic2*, major QTL, maize (*Zea mays*), mild mutation, plant height, rare allele.

## Abstract

Plant height has long been an important agronomic trait in maize breeding. Many plant height QTLs have been reported, but few of these have been cloned. In this study, a major plant height QTL, *qph1*, was mapped to a 1.6kb interval in *Brachytic2* (*Br2*) coding sequence on maize chromosome 1. A naturally occurring rare SNP in *qph1*, which resulted in an amino acid substitution, was validated as the causative mutation. *QPH1* protein is located in the plasma membrane and polar auxin transport is impaired in the short near-isogenic line RIL88_(qph1)_. Allelism testing showed that the SNP variant in *qph1* reduces longitudinal cell number and decreases plant height by 20% in RIL88 _(qph1)_ compared to RIL88 _(QPH1)_, and is milder than known *br2* mutant alleles. The effect of *qph1* on plant height is significant and has no or a slight influence on yield in four F_2_ backgrounds and in six pairs of single-cross hybrids. Moreover, *qph1* could reduce plant height when heterozygous, allowing it to be easily employed in maize breeding. Thus, a less-severe allele of a known dwarf mutant explains part of the quantitative variation for plant height and has great potential in maize improvement.

## Highlight

A rare SNP mutation in *Brachytic2* underlies a major QTL affecting plant height, and moderately reduces plant height and increases yield potential in maize.

## Introduction

Short stature, erect leaf angle, disease resistance, and high yield are traits that have been pursued by breeders for decades. Cereal production sharply increased in the 1960s as the ‘Green Revolution’ popularized the use of dwarf and semi-dwarf cultivars. Likewise, maize (*Zea mays*) production improved dramatically due to the adoption of hybrids and use of moderately short varieties that are more resistant to lodging and compatible with higher planting density (Duvick, 2005). Reduced ear height and an increased plant height/ear height ratio also have the potential to increase dry matter accumulation (Yang *et al.*, 2010a). Many of the Green Revolution genes, such as *sd-1* in rice (Sasaki *et al.*, 2002) and *rht1* in wheat (Peng *et al.*, 1999), have been identified and utilized in crop improvement. These genes encode proteins that either regulate the synthesis of plant hormones or modulate their signalling pathways. Several genes that strongly influence plant height and are qualitatively inherited have also been cloned in maize (Winkler and Helentjaris, 1995; Thornsberry *et al.*, 2001; Multani *et al.*, 2003); however, these mutants have not found applications in maize breeding due to their adverse impact on grain yield. Identification of alleles moderately reducing plant height is highly desirable.

Plant height QTLs are favourable candidates as mild height modulators. A number of such QTLs have been reported in cereal crops but only a few of these have been cloned (Xue *et al.*, 2008; Yan *et al.*, 2011; Teng *et al.*, 2012). The large genome size of maize makes QTL cloning a time-consuming task, although some progress has been made (Wang *et al.*, 2005; Salvi *et al.*, 2007; Zheng *et al.*, 2008). Moreover, planting density affects the grain yield of maize more than other members of the grass family (Vega *et al.*, 2001). In the past few decades, maize yield has been increased mainly by adopting modern hybrids that are more tolerant to high planting density and other biotic or abiotic stresses, and have higher efficiency in resource usage (Liang *et al.*, 1992; Vafias *et al.*, 2006). Due to competition for light, plant height increases with planting density. This tends to decrease stalk diameter and increase the potential of stalk lodging, leading to yield losses (Park *et al.*, 1989; Hashemi *et al.*, 2005). Modifying plant height and other architecture components, while not influencing grain yield, is among the key factors in developing cultivars for compact planting.

We previously identified a major QTL affecting multiple traits in bin7 on chromosome 1 using F_2:3_ (a series of F_3_ families derived from F_2_ individuals)_,_ immortalized F_2,_ and recombinant inbred line (RIL) populations derived from Zong3 and 87-1, a hybrid known as ‘Yuyu22’ that has been widely planted in China for the past two decades. This QTL, designated *qph1*, was mapped near marker umc1122 (chromosome 1: 201677909- 201678070) and explains ~10% of the phenotypic variation for plant height and ear height in the RIL, immortal F_2_, and F_2:3_ population of Zong3/87-1. *qph1* was significantly associated with plant height and marginally affects yield and yield components (Yan *et al.* 2003; Yan et al, 2006 Tang et al, 2007a, b; Ma et al, 2007; Tang et al, 2010). The objectives of this study were to fine map and clone *qph1* and to evaluate its use in maize breeding.

## Materials and methods

### Mapping population of *qph1* and near-isogenic lines

A RIL population of 294 lines was constructed previously from a Zong3×87-1 cross. Two RILs, RIL88_(qph1)_ and RIL279_(QPH1),_ which differed in plant height but shared a large portion of the same genetic background, were chosen to generate the near-isogenic line RIL88_(QPH1),_ with the *QPH1* allele in a RIL88 background (Supplementary Figure S1). RIL88_(qph1)_ was used as the recurrent parent to backcross RIL279 and plants were selected based on phenotype (tall plants were kept) in each generation until BC_4_F_1_. Ninety-four BC_4_F_1_ individuals were screened by 101 SSR markers all over the maize genome and a tall line with the *QPH1* allele, 05YH175-2, was selected on the basis of having the smallest segments introgressed from RIL279 on chromosome 1. 05YH175-2 was then selfed repeatedly to produce BC_4_F_3_ and its progeny, 08YB036-7, was selected on the basis of containing a single segment of RIL279 on chromosome1 exclusively in RIL88 background. 08YB036-7 was then used to generate BC_5_F_2_ and BC_6_F_2_ fine-mapping populations, and to generate the near-isogenic line of *QPH1* (Supplementary Figure S12). 8030 BC_6_F_2_ kernels were chipped and genotyped with two flanking markers, umc2396 and MHC412 (Supplementary Table S5).

### F_2_ populations and single-cross F_1_ hybrids

Four F_2_ populations, 4F1×81162, Ye107 × Zheng32, Ye107 × B73, and Zong3 × Chuan48-2, were generated. Two markers closely linked to *qph1*, umc2396 and MHC412, were used for genotyping (umc2396 for the Zong3/Chuan48-2 F_2_ population and MHC412 for the other three F_2_ populations). A small piece of each F_2_ kernel was chipped and genotyped using the soda boiling DNA extraction method (Gao *et al.*, 2008) before planting; more than 50, 100, and 50 seeds in the *QPH1/QPH1*, *QPH1/qph1*, and *qph1/qph1* genotype classes were planted and phenotyped, respectively. Zong3, 81162, Ye107, W138, B73, and Chang7-2 were crossed with RIL88_(qph1)_ and RIL88_(QPH1)_ to generate six pairs of hybrids; >50 seeds were planted and analysed in each genotype class. Two replicates of the F_2_ population and hybrids were planted to collect data for plant height, ear height, and yield components. Plots were designed with 50 rows per plot and 13 individuals per row.

### *br2* mutant-derived populations

The *br2* mutants 117A, 114F, 114G, and 121B were provided by MGCSC (Maize Genetics Cooperation Stock Centre); 117A carries the *Hahn6* allele of *br2* (Leng and Vineyard, 1951). 114G and 114F are linkage stocks from two different sources that each carried an unspecified *br2* mutant; these two lines also have *hm1*, *hm2* mutations besides the *br2* mutation (http://www.maizegdb.org). 121B was originally named *mi8043* and was found to be an allele of the *br2* gene (Marty Sachs, personal communication). 117A, 114F, 114G, and 121B were crossed with RIL88_(qph1)_ and RIL88_(QPH1)_ to develop four pairs of single-cross hybrids. 117A × RL88_(qph1)_ was selfed to generate an F_2_ population; umc2396 was used for genotyping. Plant height and ear height data were obtained for 36, 89, and 54 individuals in *br2-117A/br2-117A*, *qph1/br2-117A*, and *qph1/qph1* genotype classes, respectively. Plant height and ear height of individuals in these populations were measured and analysed. Plot design was the same as used for the four F_2_ populations and six pairs of single-cross hybrids.

Dwarf line N546 is derived from Mexican super dwarf (Johnson *et al.*, 1998). Nine crosses, 93NEX501 × PHVRZ, 93NEX501 × PHHHN, 93NEX501 × PHVNV6, Y93NEX504 × PHHHN, YN546 × PHF0D, YN546 × PHCCW, YN546 × PHVRZ6, YN546 × PHVNV, and YN546 × PH128S were made between parents carrying the *br2-bj* allele (the former) and normal elite inbred lines (the latter). F_2_ individuals were genotyped with markers PZE-101155635 and PZE-100001759, which are adjacent to *br2-bj*, and phenotyped for plant height and ear height (Ganal *et al.*, 2011).

### DNA preparation and sequence analysis

DNA was extracted from young seedlings using the CTAB method (Dellaporta *et al.*, 1983). For BC_6_F_2_ mapping population and the four F_2_ populations, DNA was extracted from chipped kernels using the soda boiling DNA extraction method (Gao *et al.*, 2008). The genomic sequences of *qph1* and *QPH1* were amplified with H109F/H106R, H114F/R, H103F/R, H212F/233R, and H115F/116R. The *Br2-bj* allele was amplified with HF1F/R and HF2F/R. Primer sequences are listed in Supplementary Table S5. PCR was performed with Phusion High Fidelity Master Mix with HF buffer (Thermo Fisher Scientific, Pittsburgh, PA, USA) according to standard protocol. PCR products were ligated into the T-easy vector and colonies containing the desired PCR fragment were picked and sequenced; multiple colonies for each PCR product of each recombinant were sequenced and analysed to eliminate PCR errors.

### Scanning electron microscopy

The second and sixth internodes at the mid-elongation stage (15 expanded leaves and 19 visible leaves) and the uppermost internodes at the adult stage (19 expanded leaves) of RIL88_(qph1)_ and RIL88_(QPH1)_ were subjected to scanning electron microscopic examination. Stem tissues from corresponding internodes of RIL88_(qph1)_ and RIL88_(QPH1)_ were cut into 1mm longitudinal and transverse sections and fixed in FAA (Formalin:acetic acid:70% ethanol, 1:1:18, v/v/v). Fixed samples then went through dehydration with a series of graded ethanol (15min in 70%, 80%, 90%, and 100% ethanol). Samples were then treated with isoamyl acetate for 15min twice to replace the remaining ethanol and subjected to critical point drying (HITACHI, HCP-2). Dried samples were mounted on a suitable working stage and coated with Pt using a high vacuum (Eiko IB.3, ION COATER). Scanning electron microscope HITACHI S-3400N was used for imaging.

### Subcellular localization of *QPH1*

*QPH1* coding sequence was amplified by PCR using primer pair H235/GFP-R and cloned into a GFP vector with the 35S promoter to express the *QPH1–GFP* fusion protein. Plasmid containing the *QPH1–GFP* construct was transformed into onion epidermal cells through gene gun bombardment (Bio-Rad PDS-1000). Transformed cells were then incubated in 1/2 MS media for 20h at 28°C and examined by confocal laser scanning microscopy (Nikon EZ-C1).

### Expression analysis

Stem tissues of RIL88_(qph1)_ and RIL88_(QPH1)_ in three stages, at the beginning, middle, and end of elongation, was collected. In each developmental stage, 10 biological replicates of RIL88_(qph1)_ and RIL88_(QPH1)_ were sampled. Total RNA was extracted with Trizol reagent (Invitrogen, Carlsbad, CA, USA) and complementary DNA was synthesized using the AMV reverse transcription system (Promega, Madison, WI, USA) with Oligo (dT) primer. RT12F/R were used to amplify *QPH1* (*qph1*) using SYBR Premix Ex Taq^TM^ (TAKARA, Shuzo, Kyoto, Japan), with *β–actin1* as the endogenous control. Real-time PCR was performed with Real Time PCR system 7500 (Applied Biosystems) using the 2^-ΔΔCT^ method (Livak and Schmittren, 2001) according to the standard procedure.

### Sequence analysis in diverse maize lines and teosinte accessions

The association panel with 527 inbred lines used in this study is described by Yang *et al.* (2010b). The 1.4kb *qph1* target region containing all five SNPs was amplified with primers 5N3F and 211R. The 839bp SNP5259-containing region was amplified with 212F and 211R from 192 teosinte accessions. The CIMMYT genebank represented a major portion of the diversity present in the teosinte collection.

### Gene transformation in *Arabidopsis* T-DNA insertional mutant *atpgp1-2*

The *Arabidopsis* mutant SAIL_716_H02 (Columbia ecotype), designated *atpgp1-2* (At2g36910), was obtained from ABRC (Ohio State University, Columbus, OH) and genotyped with primers 716F, 716R, and LB2 for the homozygous mutant according to standard procedure (http://signal.salk.edu/tdnaprimers.2.html). Coding regions of maize *qph1* and *QPH1* alleles were amplified with primer pair 232F/233R. Site-directed mutagenesis was performed on *qph1* coding sequence to change the T to G (wild-type genotype). *QPH1*, *qph1*, and *mqph1* (the mutated *qph1*) were overexpressed using PBI121 under the CaMV 35S promoter in the *atpgp1-2* mutant. Transgenic plants were selected with kanamycin (50mg l^–1^). Plant height was measured at 28 days (16/8 light/dark photoperiod, 21–23°C). Coleoptile length for 7-day-old plants grown on 1/2MS solid medium was measured with ImageJ software (National Institutes of Health, http://imagej.nih.gov/ij) with the pictures of the plants taken by digital camera.

### Polar auxin transport measurements

Auxin transport assays were performed using the protocol described by Li *et al.* (2007) and Multani *et al.* (2003) with some modifications. Ten replicates of RIL88_(qph1)_ and RIL88_(QPH1)_ seeds were grown in sand for 5 days and harvested for coleoptiles in length of 3cm. Coleoptile segments were equilibrated in1/2MS (pH 5.8) liquid media for 2h and the apical portion was submerged in 1/2 MS solid medium containing 0.35% phytogel, 500nM unlabelled IAA (Sigma-Aldrich), and 500nM [3H]-labelled IAA (specific activity 20 Ci mmol^–1^; American Radiochemical, St Louis, MO, USA) and incubated in the dark for 5h at 25°C. Coleoptiles were then cut into 0.5cm sections and washed twice with 1/2 MS liquid medium. Washed coleoptile sections were incubated in 1ml scintillation fluid for 16h and counts were made with a scintillation counter (Perkin-Elmer MicroBetaTriLux1450).

## Results

### Effects of *qph1* on plant height and other traits

**Fig. 1.**
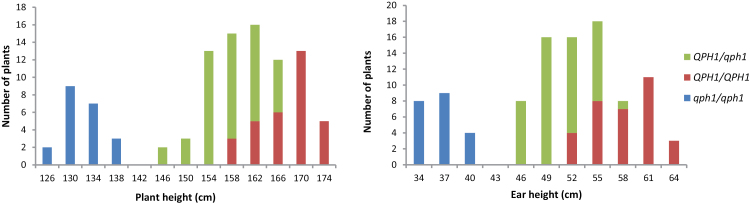
Plant height and ear height variation in BC_4_F_2:3_. Blue, green and red bars represent the number of *qph1/qph1*, *QPH1/qph1*, and *QPH1/QPH1* individuals in the BC_4_F_3_ population developed from RIL88 and RIL279. Plant height (A) and ear height (B) segregations are consistent with the segregation of a single Mendelian factor.

To identify the underlying gene for QTL *qph1*, RIL88_(qph1)_, and RIL279_(qph1)_, two lines carrying *qph1* and *QPH1* alleles, respectively, and sharing a large proportion of the same genetic backgrounds, were used to generate a BC_4_F_2_ fine-mapping population (Supplementary Figure S1). The previously identified plant height QTL *qph1* was further mapped between markers umc1035 (chromosome 1: 195219753-195219900) and umc2236 (chromosome 1: 198266651-198266734) on chromosome 1 in BC_4_F_2_ (Supplementary Figure S2). In BC_4_F_2:3_, the numbers of *qph1/qph1*, *qph1/PQH1*, and *QPH1/QPH1* individuals were 21, 42, and 32; segregation of tall to short plants was ~3:1 for both plant height and ear height (*χ* ^2^ = 0.196, *P* > 0.05), indicating the presence of a single recessive Mendelian factor (Fig. 1). In contrast, flowering time and yield components displayed continuous variation (Supplementary Figure S3), consistent with the hypothesis that *qph1* has major effects on plant height and ear height and minor effects on other traits. Plant height and ear height differences were highly significant with *P*-values of 9.30E-48 and 3.40E-44 (*t*-test) with the dominant allele of *QPH1* contributing 90% to plant height and 87% to ear height. Differences in days to silking and days to tasseling were also marginally significant (*P* = 0.0303 and 0.0002, respectively), revealing that the *QPH1/QPH1* individuals flower slightly earlier. Although ear length, diameter, and weight were all significantly different (*P* = 0.0002, 9.50E-05 and 9.00E-08), the effects of *qph1* on these yield components were relatively small (Table 1).

**Table 1.** Analysis of the agronomic traits in BC4F2:3 (2007, Beijing)

	*qph1/qph1*	*QPH1/QPH1*	*QPH1/qph1*	A	D	D/A	R^2^ (%)	*P*-value
PH	130.5±3.3	165.9±4.7	156.2±4.8	17.7	7.8	0.4	90	9.30E-48
EH	35.4±1.9	57.2±3.4	49.6±3.1	10.9	3.3	0.3	87	3.40E-44
ED	4.3±0.1	4.4±0.1	4.4±0.1	0.1	0	0	17	9.50E-05
EL	13.8±0.4	14.4±0.6	14.2±0.5	0.3	0.2	0.5	16	0.0002
EW	58.9±9.3	74.9±9.8	67.5±8.8	8	0.6	0.1	28	9.00E-08
DTS^1^	90.7±1.0	90.4±1.4	89.6±1.0	–0.2	–1	6.6	16	0.0002
DTS^2^	93.2±0.8	92.9±1.4	92.5±0.9	–0.2	–0.6	3.5	7	0.0303
N	21	32	46					

PH, plant height; EH, ear height; ED, ear diameter; EL, ear length; EW, ear weight; DTS^1^, days to shedding; DTS^2^, days to silking. Data are expressed as mean ± SD.

### Morphological and cytological observations

**Fig. 2.**
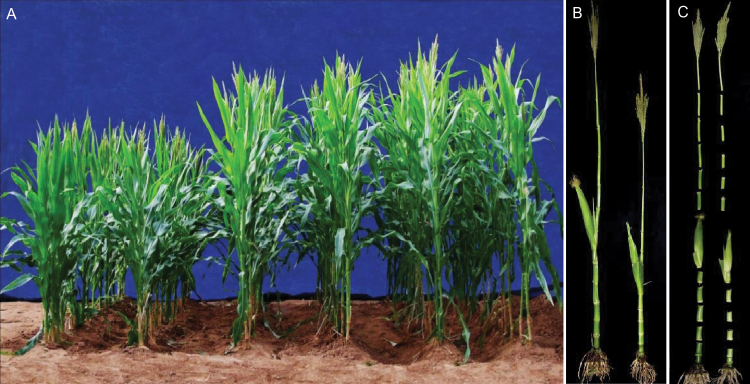
Plant height and internode length of RIL88_(QPH1)_ (BC_6_F_3_) and RIL88_(qph1)_. (A) RIL88_(qph1)_ (two rows on the left), RIL88_(QPH1)_ (two rows on the right), and their F_1_ hybrid (two rows in the middle). Plant height of the hybrid is between the two parental lines and slightly shorter than RIL88_(QPH1)_. (B) Plant height and ear height comparison between RIL88_(QPH1)_ (left) and RIL88_(qph1)_ (right). (C) Internode length comparison between RIL88_(QPH1)_ (left) and RIL88_(qph1)_ (right); a greater decrease in internode length from the top of the plant down is observed between near-isogenic lines.

**Fig. 3.**
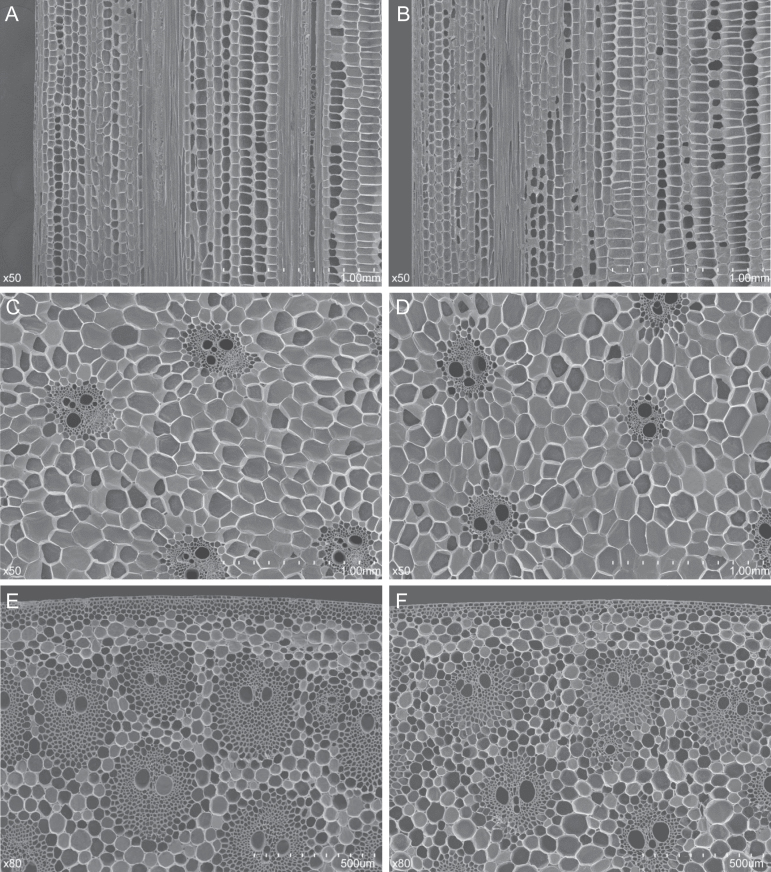
Scanning electron microscopy examination of the second internodes from RIL88_(qph1)_ and RIL88_(QPH1)_. The second internodes of near-isogenic lines in mid-elongation stage were subjected to histological analysis. (A, B) Longitudinal view of the parenchyma cells of RIL88_(QPH1)_ and RIL88_(QPH1)_. (C, D) Transverse view of the parenchyma cells of RIL88_(qph1)_ and RIL88_(QPH1)_. (E, F) Transverse view of the epidermal region of RIL88_(qph1)_ and RIL88_(QPH1)_.

To study the effect of *QPH1* and *qph1* alleles on plant height and its components in the RIL88 background, RIL88_(QPH1)_ (BC_6_F_3_), which only has a 5kb segment of RIL279 introgressed into RIL88 background, was generated (Supplementary Figure S1). Plant height exhibited an incomplete recessive (partial dominant) effect (Fig. 2a). Plant height variation between RIL88_(QPH1)_ and RIL88_(qph1)_ was affected by both average internode length and difference in internode number. RIL88_(QPH1)_ had one more internode below the ear; however, plant height difference was contributed mostly by internode length difference, especially below the ear (Fig. 2BC). Moreover, a gradual increase in internode length difference from the top of the plant down was observed; the uppermost internode showed no difference in length between the near-isogenic lines while the second lowest internode of RIL88_(qph1)_ was more than 50% shorter than wild type (Supplementary Figure S4). The short stature of *qph1* was similar to other *br2* mutants but with a milder phenotype (Scott *et al.*, 1969). Cytological analysis was performed on the second, sixth, and top internodes of RIL88_(QPH1)_ and RIL88_(qph1)_ through scanning electron microscopy, statistical data on cell length, cell number per mm, and calculated longitudinal total cell number per internode is summarized in Supplementary Table S1. No significant difference was detected between RIL88_(QPH1)_ and RIL88_(qph1)_ for either cell length (*P* = 0.71, 0.43, and 0.48) or number of cells per mm (*P* = 0.33, 0.23, and 0.52) in the second (Fig. 3), sixth (Supplementary Figure S5) and top internodes (Supplementary Figure S6). This indicates that the length difference was caused by the different longitudinal cell number between near-isogenic lines rather than different cell size (Supplementary Table S1). Furthermore, altered structure of the vascular bundles in epidermal regions of the second and sixth internodes was observed between RIL88_(QPH1)_ and RIL88_(qph1)_ (Fig. 3E, F; Supplementary Figure S5); however, the uppermost internodes do not show cell number differences or differences in epidermal vascular structure (Supplementary Table S1; Supplementary Figure S6), indicating that the reduced longitudinal cell number is related to the short stature of RIL88_(qph1)._

### Map-based cloning of *qph1* and validation through allelism testing

**Fig. 4.**
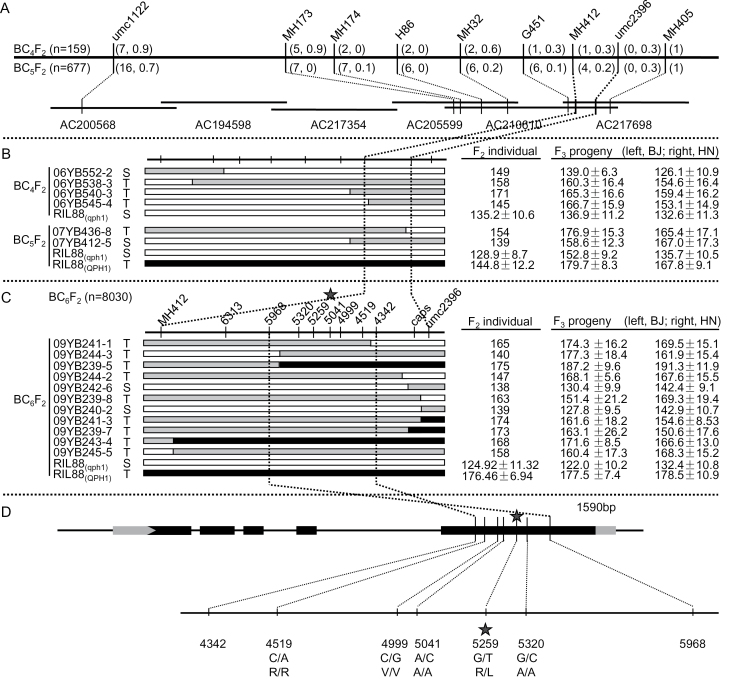
Map-based cloning of *qph1.* (A) Locations of markers used in BC_4_F_2_ and BC_5_F_2_ populations. Number of recombinants and crossover rates between markers are shown before and after the comma. (B) Recombinants identified in BC_4_F_2_ and BC_5_F_2_. White, black, and grey indicate homozygous RIL88_(qph1)_, homozygous RIL88_(QPH1)_, and heterozygous genotypes. Plant height of recombinant individuals and their progenies in two locations are shown. T, tall; S, short. (C) Fine mapping of *qph1* using the BC_6_F_2_ population. MHC412 and umc2396 were used as flanking markers to identify recombinants. Recombinants were sequenced to determine crossover break points, allowing the *qph1* interval to be narrowed down to 4.9kb between SNPs 5968 and 4342. (D) Sequence analysis of the *qph1* target region. The *qph1* interval locates in exon 5 of the *Br2* gene. Five SNPs were identified within it; only SNP5259 caused an amino acid substitution from arginine to leucine. Nucleotides and amino acids in RIL88_(qph1)_ and RIL88_(QPH1)_ for polymorphic loci are shown before and after the slash.

Two lines, RIL279 (the tall line and donor of allele *QPH1*) and RIL88 (the short line and recurrent parent, containing the *qph1* allele), which shared 70% genetic background, were selected from the Zong3/87-1 RIL population to generate a fine-mapping population of *qph1*. The *qph1* allele was first mapped to a 4.9kb region flanked by umc2396 (chromosome 1: 202337847-202337976) and MH412 (chromosome 1: 202342789-202342908) on BAC AC210610 in BC_4_F_2_ and BC_5_F_2_ populations (Fig. 4AB). To achieve higher map resolution, an expanded BC_6_F_2_ mapping population was developed and out of the 8030 individuals genotyped from kernel chips, 45 recombinants of 11 crossover types were identified by genotyping with flanking markers umc2396 and MH412 (Fig. 4C). Eleven recombinants were sequenced for the five SNPs within the target region, and the progeny plants were phenotyped in two different locations (Beijing and Hainan). Two key recombinants, 09YB241-1 and 09YB244-3, allowed the mapping interval to be narrowed down to 1590bp. The final interval has both borders falling in the fifth exon of the maize *Brachytic2* gene (*ZmPGP1*, GRMZM2G31537), and among the five identified SNPs, only SNP5259 (G/T) caused an amino acid substitution from arginine (R) to leucine (L) (Fig. 4D).

To evaluate the allelic effect between the natural variation present and the known *br2* mutants, four *br2* mutants (114F, 114G, 117A, and 121B) were obtained and crossed with RIL88_(QPH1)_ and RIL88_(qph1)_, respectively. Significant differences in plant height and ear height between the hybrids were observed; the phenotypic defects of the four *br2* mutants were completely compensated by crossing with RIL88_(QPH1)_ and only partially reversed by crossing with RIL88_(qph1)_ (Supplementary Figure S7), implying that *qph1* is allelic to the *Br2* gene and is defective compared with *QPH1*. Moreover, in the F_2_ population of 117A and RIL88 _(qph1)_, plant height and ear height segregated in a 3:1 ratio (*qph1/qph1* and *qph1/br2-117A* plants to *br2-117A/br2-117A* plants), indicating that the *qph1* allele is dominant to *br2-117A* (Supplementary Figure S7). Moreover, although RIL88_(qph1)_ is not as short as the four *br2* mutants, it has comparably low ear height (Supplementary Figure S7), which is a favourable feature directly related to lodging resistance.

### Subcellular localization of *QPH1*

In order to detect the subcellular location of *QPH1*, the full-length *QPH1* protein fused at its C-terminus with the GFP coding region protein was overexpressed in onion epidermal cells using the GFP construct not fused to a plant protein as the control. As revealed by transient expression results, the untargeted GFP was expressed throughout the whole transformed cell in cytoplasm, nucleus, and membrane. However, the *QPH1–GFP* fusion protein was expressed exclusively in membrane (Supplementary Figure S8); this result is consistent with the known localization of *br2* homologues and cellular function of ABC transporters.

### Expression analysis

The *Br2* gene was previously shown to have high expression in stalk internodes, moderate expression in leaves, and very low expression in roots (Mutani *et al*., 2003). To investigate the underlying mechanism for the reduction in internode length of RIL88_(qph1),_ stem tissues of RIL88_(qph1)_ and RIL88_(QPH1)_ from three developmental stages during elongation, when the phenotypic difference in internode length starts to be visible, were subjected to qRT-PCR analysis. The expression of *qph1* was lower in period A at the beginning of elongation, reached a higher level in mid-elongation at period B, and began to decrease in period C before the end of elongation. T-test showed no significant differences in expression between RIL88_(qph1)_ and RIL88_(QPH1)_ in any of the three stages, indicating that the plant height and ear height difference between the near-isogenic lines is not caused by the difference in transcription level of *qph1* (Supplementary Figure S9).

### Association analysis using the *qph1* SNPs

Based on the association result of plant and ear height in a population consisting of 527 inbred lines (Yang et al, 2010b), SNP5259 is a rare SNP that only exist in five lines and the four synonymous SNPs (SNP4519, 4999, 5041, and 5320) were shown not to be associated with phenotype. Due to the low number of inbred lines that have nucleotide T at position 5259, the non-synonymous SNP5259 could not be validated by association analysis (Supplementary Table S2). The five inbred lines that harbour SNP5259 (T), 81162, Ye107, Dan9046, W138, and Zong3 are all temperate lines that belong to the same heterotic group. All five lines have plant heights shorter than average: 113, 144, 133, 136, and 153cm for 81162, Ye107, Dan9046, W138, and Zong3, respectively. (Data was collected from >50 plants grown in five different locations; the average plant height of 527 inbred lines was 173cm.)

A total of 192 teosinte entries (data not shown) were also sequenced and analysed for the *qph1* confidence interval. The four synonymous SNPs were found existing in either homozygous or heterozygous states in teosinte; however, only the homozygous G allele (wild-type genotype) was identified for SNP5259, suggesting that the causative mutation in *qph1* most likely occurred as part of the temperate maize breeding program after the domestication of maize. The rare frequency of SNP5259 (T) also implies that the mutation occurred very recently and is not widely used in breeding programs.

### Validation of *qph1* and its functional site with *Arabidopsis* mutant *atpgp1-2*

**Fig. 5.**
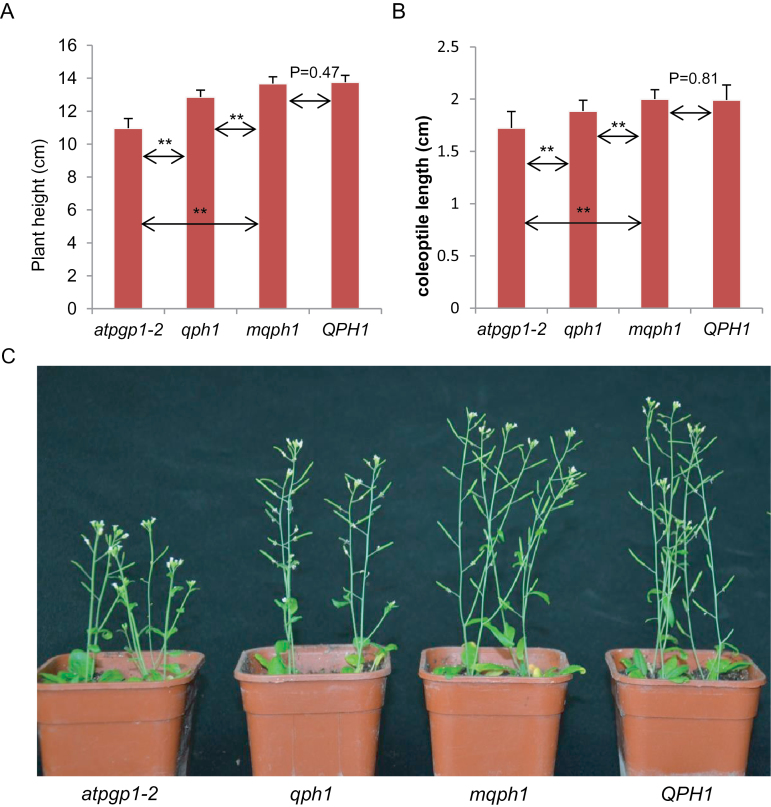
Validation of *qph1* and its functional site through transformation of *Arabidopsis* homologous mutant *atpap1-2.* (A) Plant height of the *atpgp1-2* mutant transformed with pBI121 empty vector, *qph1*, *mqph1* (mutated *qph1*), and *QPH1*. (B) Coleoptile length of the *atpgp1-2* mutant transformed with pBI121 empty vector, *qph1*, *mqph1* (mutated *qph1*), and *QPH1*. (C) Plant height of *qph1*, *mqph1* (mutated *qph1*), and *QPH1* transformation plants. **, significant difference (0.01 level). Data is expressed as mean ± SD.

To validate the function of *qph1* and its functional site SNP5259, site-directed mutagenesis was performed on the maize *qph1* allele to mutate SNP5259 (T) to SNP5259 (G). *QPH1*, *qph1*, and *mqph1* (mutagenized *qph1*) were cloned into vector PBI121 and overexpressed with the 35S promoter in *Arabidopsis* T-DNA insertional mutant *atpgp1-2* (*AtPGP1*, At2g36910, is the homologue of the maize *Br2* gene in *Arabidopsis*), which has reduced plant height and coleoptile length (Ye et al, 2013). *QPH1* and *mqph1* could restore the plant height and coleoptile length of the *atpgp1-2* mutant to that of the wild type; in contrast, transformation of *atpgp1-2* with the *qph1* allele led to an intermediate level of rescue (Fig. 5). No significant difference was observed between *QPH1* and *mqph1* transgenic plants in plant height and coleoptile length, but a significant difference was detected between *qph1* and *mqph1* transgenic plants (*P* = 0.0007 for plant height and *P* = 0.005 for coleoptile length) (Fig. 5AB); the only difference between *mqph1* and *qph1* coding sequence is the G/T polymorphism at SNP5259; this result is consistent with the hypothesis that SNP5259 (T) is the underlying causative mutation in *qph1*. Moreover, statistical analysis showed that the difference in plant height and coleoptile length between *qph1* transgenic plants and the *atpgp1-2* control also reached significant levels (*P* = 0.00023 and 2.93E-11), suggesting that *qph1* is not a complete loss of function allele (Fig. 5A, B).

### Polar auxin transport in RIL88_(qph1)_, RIL88_(QPH1)_ and the *br2* mutant 114F

**Fig. 6.**
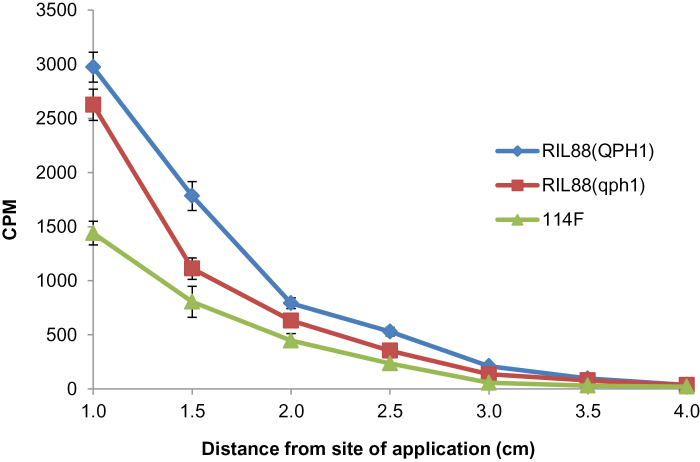
Transport of [^3^H] IAA in coleoptiles of RIL88_(QPH1)_, RIL88_(qph1)_, and the *br2* mutant 114F. CPM, counts per minute. Error bars indicate SD, n=8.

Because the maize *Br2* gene is known to function in polar auxin transport (Multani et al, 2003), an assay of basipetal transport of [^3^H] IAA was carried out in the coleoptiles of RIL88_(qph1)_, RIL88_(QPH1)_, and the null *br2* mutant 114F. Results showed that [^3^H] IAA translocation to the lower sections of the coleoptiles was significantly reduced in RIL88_(qph1)_ and the *br2* mutant 114F compared to RIL88_(QPH1)_ (Fig. 6). As the *br2* mutant consistently showed increased loading of auxin into the upper coleoptile near the site of application in former studies (Multani et al, 2003), the difference was probably caused by the impaired polar auxin transport in defective *br2* plants. Consistent with the hypothesis that the *qph1* allele is partially defective compared with the *br2-117A* allele, a significant difference in [^3^H] IAA translocation between RIL88_(qph1)_ and 114F was detected, indicating that *qph1* was not a complete loss of function allele.

### Functional assessments of *qph1* in F_2_ populations of different genetic backgrounds

To evaluate the effect of *qph1* under different genetic backgrounds, four F_2_ populations, 4F1×81162, Ye107 × Zheng32, Ye107 × B73, and Zong3 × Chuang48-2 were constructed by crossing the three lines containing the rare SNP (T) (81162, Ye107, and Zong3) with normal inbred lines (4F1, Zheng32, B73, and Chuan48-2). The four F_2_ populations were planted in two different locations, Beijing and Hainan (200 individuals per population in each location). In each replicate, 50 *qph1/qph1*, 100 *qph1/QPH*, and 50 *QPH1/QPH1* individuals per population were phenotyped. In both locations, a significant difference in plant height and ear height was detected between *QPH1/QPH1* and *qph1/qph1* individuals and between *QPH1/qph1* and *qph1/qph1* individuals. Plant height difference between *QPH1/QPH1* and *qph1/qph1* individuals in the B73 × Ye107 F_2_ population is shown as an example (Supplementary Figure S10). Single-factor ANOVA analysis in 4F1/81162 F_2_ detected a strong dominant effect for plant height (D/A = 1.3 and 0.9, *P* = 6.06E-16 and 1.21E-18 in 09HN and 10BJ, respectively); similar results were also observed in the other three F_2_ populations. 15–49% and 4–37% phenotypic variation could be explained by *qph1* for plant height and ear height, respectively, in different genetic backgrounds and environments (Supplementary Table S3 and Supplementary Figure 7A). These results show that *qph1* has a consistent effect in reducing plant height and ear height in multiple genetic backgrounds.

A semi-dwarf mutant N546 (Johnson *et al.*, 1998) has features very similar to the *br2* recessive lines and a QTL peak for plant/ear height was detected at the *Br2* region in the N546 × PHB00 F_2_ population. The N546 *Br2* allele is designated *br2-bj*; it has a 3.5kb insertion of En/Spm-like transposon 660bp upstream of the predicted TATA box and a complete *gag/pol* retro-transposon insertion of 4.7kb in exon 5 which truncates the last 153 amino acids of the protein. Mean plant/ear height for *br2-bj*/*br2-bj* individuals are 88% and 50% of the heterozygous and wild-type plants, respectively. Consistent with the predicted reduction or loss of function of this complex allele, *br2-bj* behaves as a recessive allele in nine F_2_ populations constructed by crossing N546 and two of its conversion lines N501 and N504 (with the *br2-bj* allele) with normal lines (PHVRZ, PHHHN, PHVNV, PHF0D, OHCCW, and PH128S). In these segregating populations, heterozygous and wild-type plants showed no significant difference in plant height; ear height between wild-type and heterozygous plants showed a significant difference in some crosses depending on the background (Supplementary Figure S11). Results indicate that *br2-bj* has different effects on plant height and ear height in F_2_ segregation populations from *qph1*.

### Functional assessments of *qph1* in six pairs of single-cross hybrids

**Fig. 7.**
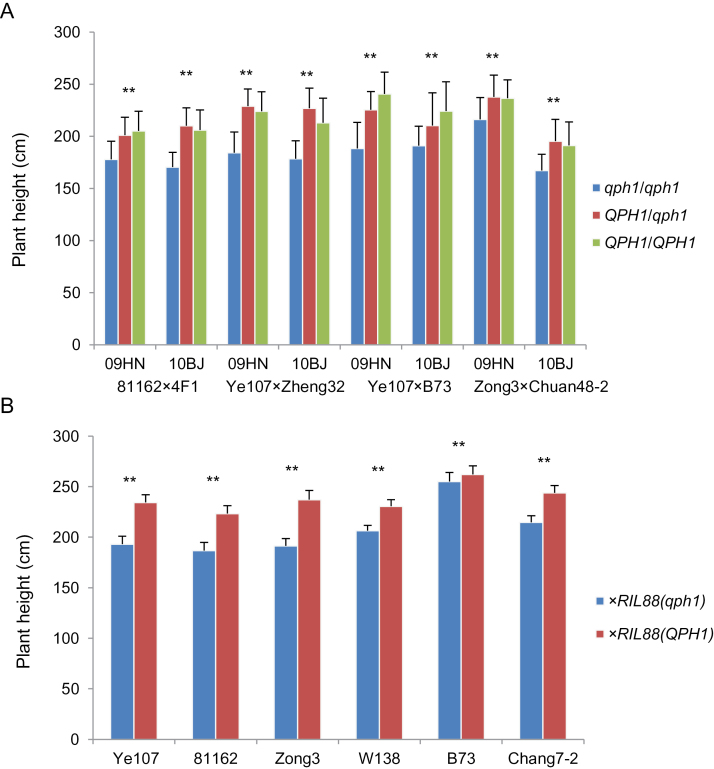
Plant height analysis of the four F_2_ populations and six pairs of single-cross hybrids. (A) Blue, red, and green represent the plant height of the *qph1/qph1*, *QPH1/qph1*, and *QPH1/QPH1* individuals, respectively. Phenotype data were collected from two replicates in Hainan (2009, left) and Beijing (2010, right). 50, 100, and 50 individuals of *qph1/qph1*, *QPH1/qph1*, and *QPH1/QPH1* were planted and analysed, respectively, for each population. (B) Plant height of RIL88_(qph1)_ (blue) and RIL88_(QPH1)_ (red) derived single-cross hybrids; 30–40 individuals were planted and analysed, respectively, in each genotype class, and data was collected from two replicates for each hybrid. **, significant difference (0.01 level). Data are expressed as mean ± SD.

Because hybrid maize is used in agriculture, the effect of *qph1* was also estimated in hybrid backgrounds. Six pairs of single-cross hybrids were generated by crossing RIL88_(QPH1)_ and SNP5259 (T)-containing RIL88_(qph1)_ with the inbred lines with or without the SNP5259 (T); effects of *qph1/qph1* with *qph1/QPH1* and *QPH1/qph1* with *QPH1/QPH1* under the same hybrid genetic backgrounds were evaluated. Plant and ear height showed significant differences between each pair of hybrids (Fig. 7B). In the hybrids derived by crossing Ye107, 81162, Zong3, and W138 with RIL88_(qph1)_, plant heights between F_1_ individuals of *qph1/qph1* were reduced by 10–24%, respectively, compared to their *QPH1/qph1* counterparts derived from RIL88_(QPH1)_. Reductions in ear height were also significant, ranging from 26 to 38%. These results indicate the strong effect of the homozygous recessive *qph1/qph1* in reducing plant height and ear height. Moreover, in B73 and Chang7-2 F_1_ hybrids, due to the incomplete recessive feature of *qph1*, significant differences in plant height and ear height between *QPH1/QPH1* and *QPH1/qph1* hybrids were also detected; phenotypic variations in plant height and ear height range from 3 to 12% for plant height and 6 to 23% for ear height, indicating that *qph1* is able to reduce plant height and ear height in a heterozygous state. *qph1* is proved to have a significant effect on plant height and ear height in hybrids, and the incomplete recessive feature of *qph1* allows the plant and ear height of a hybrid to be modified by introducing it into only one parent.

### Prospect evaluation of the impact of *qph1* on grain yield

In our previous studies, a pleiotropic effect of *qph1* causing a marginal influence on yield and yield components was detected in RIL, IF_2_, F_2:3_, and BC_4_F_2_ populations of Zong3/87-1; this was probably contributed by the genetic backgrounds (Tang et al., 2007a, b ). To evaluate the potential of *qph1* in maize improvement in terms of increasing yield potential, a number of agronomic traits of the six pairs of F_1_ and four F_2_ populations derived from *qph1*-containing lines were measured and analysed. Most yield components exhibited no significant difference between *qph1/qph1, QPH1/qph1*, and *QPH1/QPH1* individuals in the four F_2_ populations, and significant differences could be detected in only one biological repeat for a few traits in some F_2_ populations, indicating that *qph1* has no or a slight influence on maize yield under different genetic backgrounds while maintaining a shorter plant height and ear height (Supplementary Figures S10 and S12). Likewise, except for ear kernel weight and 100-kernel weight in 81162 F_1,_ and ear weight and days to shedding in Zong3 F_1_ and ear kernel weight in B73 F_1_, no additional significant difference was identified between each pair of the single-cross hybrids, suggesting that *qph1* only has a minor impact on yield under hybrid backgrounds (Supplementary Table S4). These results suggested that *qph1* could significantly reduce plant height and ear height with no or very little negative impact on yield under multiple genetic backgrounds. *qph1* could affect plant height and ear height when heterozygous, making it very useful for hybrid maize breeding.

## Discussion

### Molecular mechanism underlying the major plant height *QTL qph1*

**Fig. 8.**
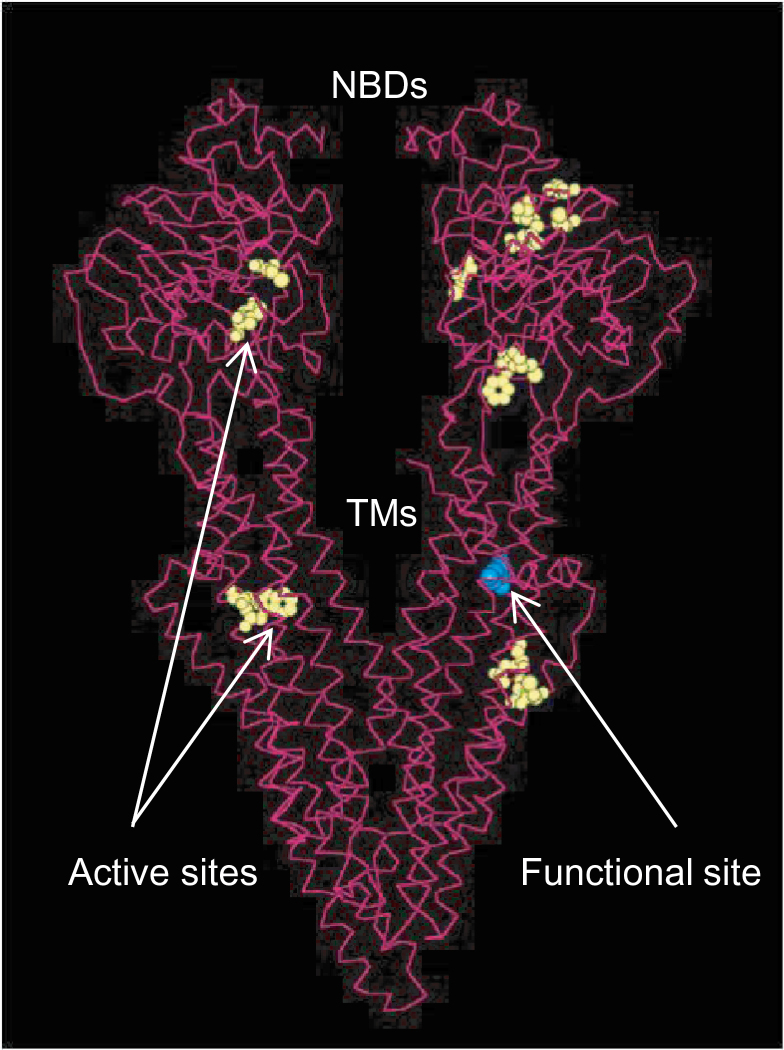
Protein structure simulation of *qph1* generated using Pymol. The six active sites of the protein are shown in yellow; the Arginine to Leucine amino acid substitution on the ninth α-helix in the transmembrane domain is indicated in blue. TMs, transmembrane domains; NBDs, nucleotide binding domains.

In this study, we report a rare SNP mutation in the maize *Brachyric2* gene underlying the major plant height QTL *qph1*. The maize *Br2* (*ZmPGP1*) gene is an ABC (ATP-binding cassette) transporter which belongs to the MDR (multi-drug resistant) class of P-glycoprotein (Noh *et al.*, 2001; Multani *et al.*, 2003) and functions in polar auxin transport as an efflux carrier. The protein molecule consists of two transmembrane domains (TMDs) that provide the translocation pathway of auxin and two cytoplasmic nucleotide-binding domains (NBDs) that hydrolyse ATP and drive the transport reaction (Chang and Roth, 2006; Aller *et al.*, 2009). The two TMDs span the membrane through the 12 α-helices (six per domain) and enable membrane insertion and regulation. The predicted causative mutation of *qph1*, SNP5259 (T), which resulted in the arginine to leucine substitution, is located on the ninth α-helix in the TMD of *Br2* and thus affects the efficiency of the transmembrane channel (Fig. 8). As an efflux carrier, amino acid residues along the transporter channel are strictly arranged (in this case, all residues are positively charged); Arg has a hydrophilic and positively charged side chain, while Leu is hydrophilic and neutral; the substantial change from Arg to Leu in *qph1* is very likely to have affected its interaction with negatively charged IAA^–^ inside the cell. Moreover, auxin is synthesized predominantly in the shoot apex, young leaves, and developing seeds (Ljung *et al.*, 2001) then dispensed to other organs by multiple efflux and influx transporters (Friml et al, 2002; Zhao *et al*, 2010). The results are consistent with the hypothesis that the defective *qph1* allele in RIL88_(qph1)_ impaired basipetal auxin transport, which led to the auxin insufficiency in lower internodes and resulted in shortened internodes. Reduced cell division and changes in vascular bundle development were observed in RIL88_(qph1)_ lower internodes, which is consistent with auxin deficiency (Galweiler *et al.*, 1998). *qph1* affects leaf number, leaf angle, and flowering time minimally compared with plant height and ear height, suggesting that it has potential for maize improvement.

### Potential application of *qph1* in maize improvement

Cereal production went through a dramatic increase due to the adoption of short cultivars during the last century, known as the Green Revolution. Underlying genes were later isolated in rice, wheat, sorghum and several other crops (Galweiler *et al.*, 1998). Obvious defects in major genes were found to be responsible for phenotypic variation in most cases, and thus made them easily manipulated for practical use. In maize, however, loss of function mutations of the major plant height genes often led to serious defects and very large yield loss (Winkler *et al.*, 1995; Thornsberry *et al.*, 2001), so moderate-effect QTLs were considered to be excellent alternatives. QTL mapping has long been conducted to localize maize plant height regulators with desirable effects, but has rarely resulted in candidate gene cloning. Several maize genes underlying quantitative traits have been cloned and validated based on linkage analysis; examples include *Vgt1*, *Tga1*, and *DGAT1-2* (Wang *et al.*, 2005; Salvi *et al.*, 2007; Zheng *et al.*, 2008), but plant height QTLs were rarely cloned (Teng et al, 2012). Among the plant height factors identified in maize so far, the recessive *br2* gene is considered to have great potential and efforts have been made to use it practically (Anderson and Chow, 1960; Djisbar and Brewabaker, 1987). Introgression of *br2* into normal varieties could reduce plant height and ear height by shortening each internode (Souza and Zinsly, 1985), but unfortunately all the recessive *br2* alleles identified so far cause severe phenotypes and it has not been possible to use them in breeding. Here, we provide the detailed phenotypic and molecular characterization of the naturally occurring mild allele of *br2*, *qph1*; it is a very rare SNP mutation that might have occurred recently and hasn’t been widely used in breeding programs. The cloning of *qph1* sheds more light on the molecular nature of natural variation at maize QTLs; it demonstrates that the naturally occurring allele at a QTL locus and a strong dwarf mutant are genetic variants of the same gene. Results of this study revealed *qph1* as a major plant height QTL that has a moderate effect on plant height and no or minimal negative effects on grain yield under various genetic backgrounds tested, suggesting its potential in maize improvement by marker-assisted selection for reduced plant height and lodging resistance.

## Supplementary material

Supplementary data can be found at *JXB* online.

Supplementary Table S1. Cytology analysis of the RIL88_(qph1)_ and RIL88_(QPH1)_ stem tissues.

Supplementary Table S2. Association analysis of the five SNPs within the target region of *qph1* in a population of 500 inbred lines.

Supplementary Table S3. Plant height and ear height analysis of the four F_2_ populations.

Supplementary Table S4. Yield-related trait analysis of single-cross hybrids derived from RIL88_(qph1)_ and RIL88_(QPH1)._

Supplementary Table S5. Primers used in this study.

Supplementary Figure S1. Construction of *qph1* fine-mapping population BC_6_F_2_ and near-isogenic lines RIL88_(QPH1)_ and RIL88_(qph1)._

Supplementary Figure S2. Fine mapping of *qph1* in BC_4_F_2_ and BC_4_F_2:3_.

Supplementary Figure S3. Phenotypic variation and distribution of the yield-related traits in BC_4_F_2:3._

Supplementary Figure S4. Stalk internode length variation between RIL88_(qph1)_ and RIL88_(QPH1)_.

Supplementary Figure S5. Scanning electron microscopy examination of the sixth internodes from RIL88_(qph1)_ and RIL88_(QPH1)_.

Supplementary Figure S6. Scanning electron microscopy examination of the uppermost internodes from RIL88_(qph1)_ and RIL88_(QPH1)_.

Supplementary Figure S7. Allelism test of *qph1*.

Supplementary Figure S8. Subcellular localization of *QPH1*.

Supplementary Figure S9. *qph1* expression in RIL88_(qph1)_ and RIL88_(QPH1)_ internodes from three developmental stages during elongation.

Supplementary Figure S10. Plant height and yield performance comparison between individuals of different genotypes in the B73 × Ye107 F_2_ population.

Supplementary Figure S11. Plant height and ear height segregation in nine F_2_ populations of N546 conversion lines and normal inbred lines.

Supplementary Figure S12. Yield component analysis of the four F_2_ populations.

## Funding

This work was supported by a grant from the National High Technology Research and Development Program of China (863 Program, No. 2012AA10A307)

## Supplementary Material

Supplementary Data
