# Supplementary material for: A rare SNP mutation in Brachytic2 moderately reduces plant height and increases yield potential in maize
Source: J Exp Bot. 2015 Apr 28;66(13):3791–802. doi: 10.1093/jxb/erv182 (PMC4473982; doi:10.1093/jxb/erv182)
Supplement: Supplementary Data [file supp_66_13_3791__index.html]

A rare SNP mutation in Brachytic2 moderately reduces plant height and increases yield potential in maize — Supplementary Data 

# A rare SNP mutation in *Brachytic2* moderately reduces plant height and increases yield potential in maize

## Supplementary Data

Data files

**Files in this Data Supplement:**

- Supplementary Data - Supplementary Data
- Supplementary Data - Supplementary Data
