# Supplementary material for: A rare SNP mutation in Brachytic2 moderately reduces plant height and increases yield potential in maize
Source: J Exp Bot. 2015 Apr 28;66(13):3791–802. doi: 10.1093/jxb/erv182 (PMC4473982; doi:10.1093/jxb/erv182)
Supplement: Supplementary Data [file supp_erv182_erv182_Sullp_figures_4.18.2015.pdf]

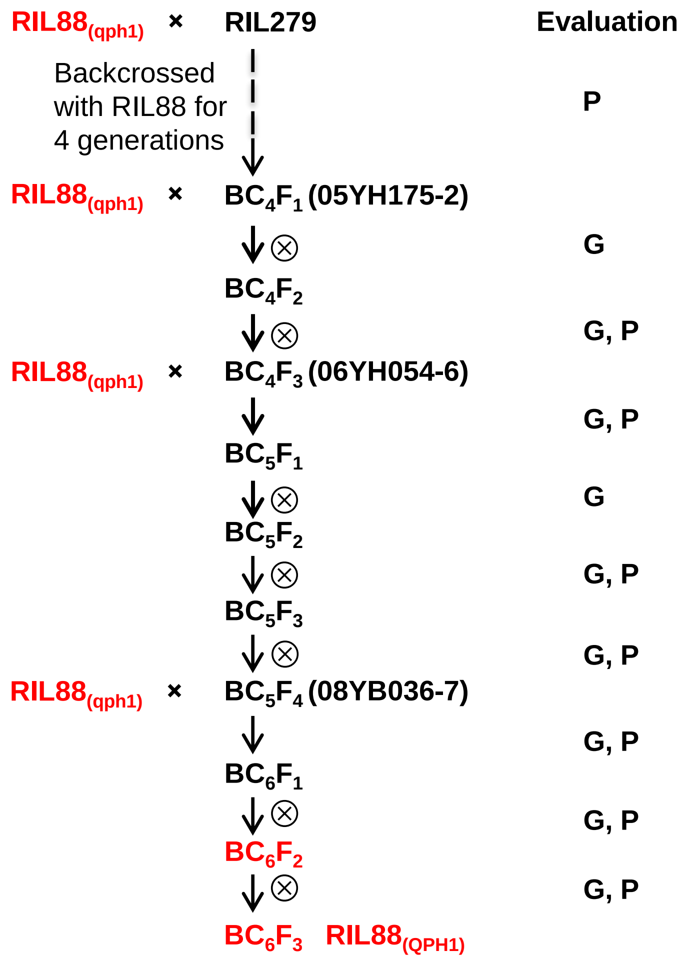

Figure S1. Construction of *qph1* fine mapping population BC_6F_2 and near-isogenic line $RIL88_{(QPH1)}$. G=evaluated by genotyping; P=evaluated by phenotyping.

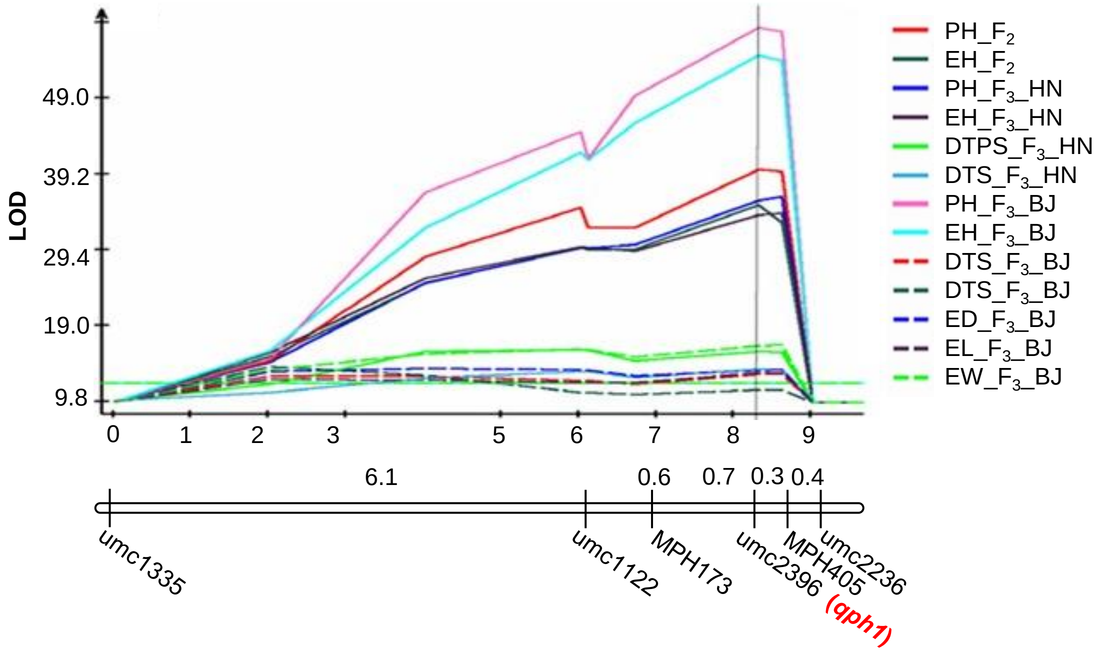

Figure S2. Fine mapping of *qph1* in BC_4F_2 and $BC_4F_{2:3}$.

qph1 was mapped to an interval between marker umc1035 and umc2236 and co-segregated with umc2396. PH=plant height, EH=ear height, DTPS=days to pollen shedding, DTS=days to silking, ED=ear diameter, EL=ear length, EW=ear weight. HN=data collected from Hainan, China. BJ=data collected from Beijing, China.

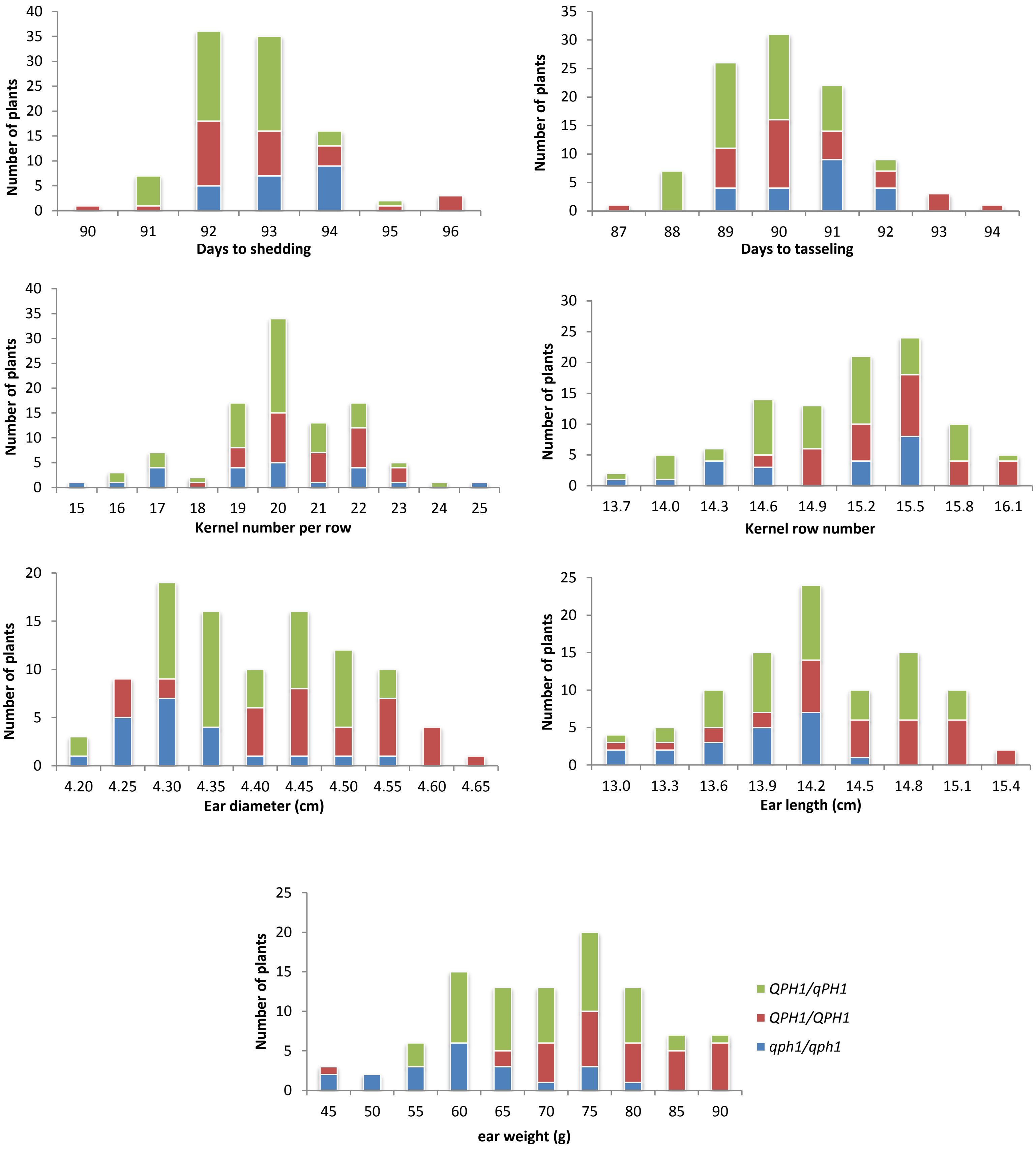

Figure S3. Phenotypic variation and distribution of yield-related traits in $BC_4F_{2:3}$.

qph1/qph1, *QPH1/qph1* and *QPH1/QPH1* plants are indicated in blue, green and red respectively, Horizontal axis stands for variation scales of different agronomic traits and vertical axis stands for numbers of plants within certain ranges.

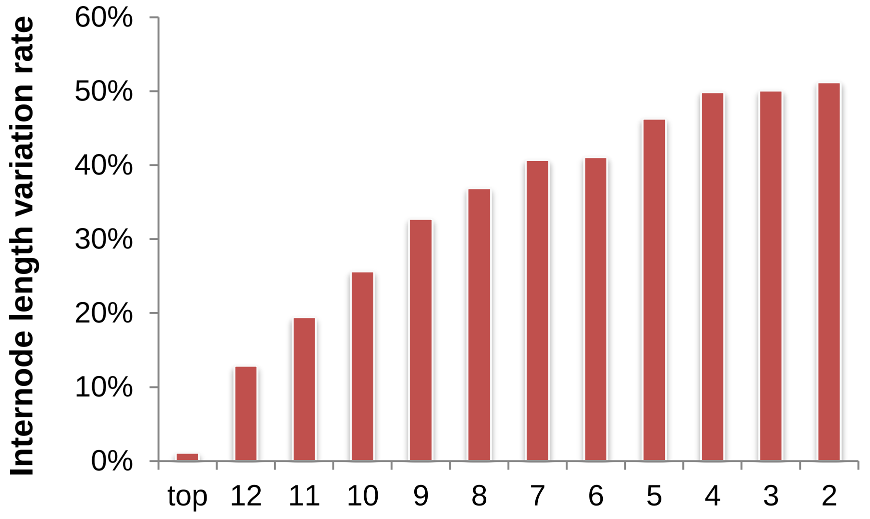

Figure S4. Stalk internode length variation between RIL88$_{(qph1)}$ and RIL88$_{(QPH1)}$。

Variation rate = [(RIL88$_{(QPH1)}$-RIL88$_{(qph1)}$)/RIL88$_{(QPH1)}$] × 100%. Internodes were counted from ground up. The uppermost internodes exhibited no significant difference in length and the variation rates gradually increase from the top of the plants towards the bottom, with the lowest internode having the greatest reduction in internode length.

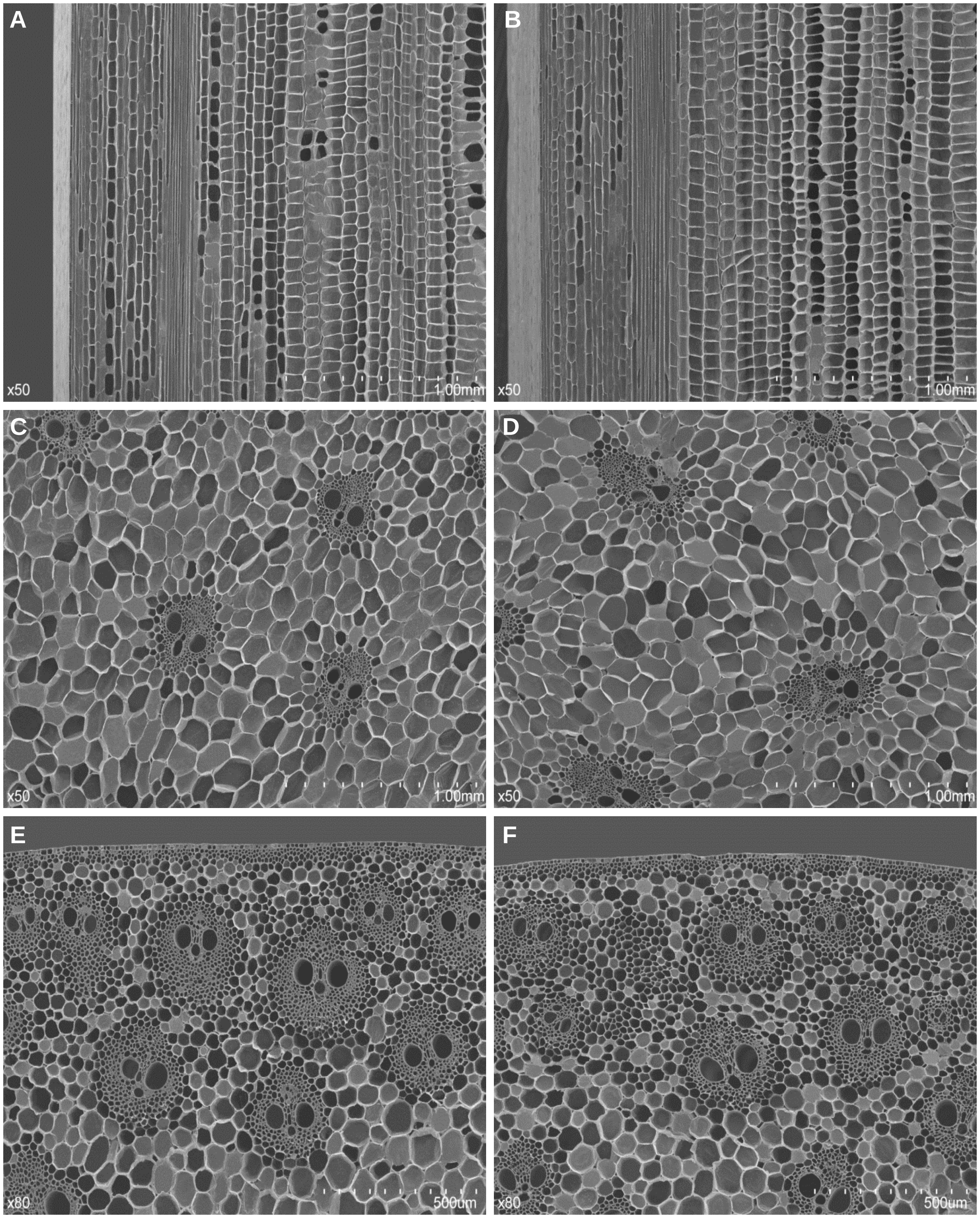

Figure S5. Scanning electron microscopy examination of the sixth internode from RIL88$_{(qph1)}$ and RIL88$_{(QPH1)}$.

The sixth internode of RIL88$_{(qph1)}$ and RIL88$_{(QPH1)}$ in mid-elongation stage were subjected histological analysis. A and B, longitudinal sections of the parenchyma cells in RIL88$_{(qph1)}$ (left) and RIL88$_{(QPH1)}$ (right); C and D, transverse view of the parenchyma cells in RIL88$_{(qph1)}$ and RIL88$_{(QPH1)}$; E and F, transverse view of the epidermal region of RIL88$_{(qph1)}$ and RIL88$_{(QPH1)}$.

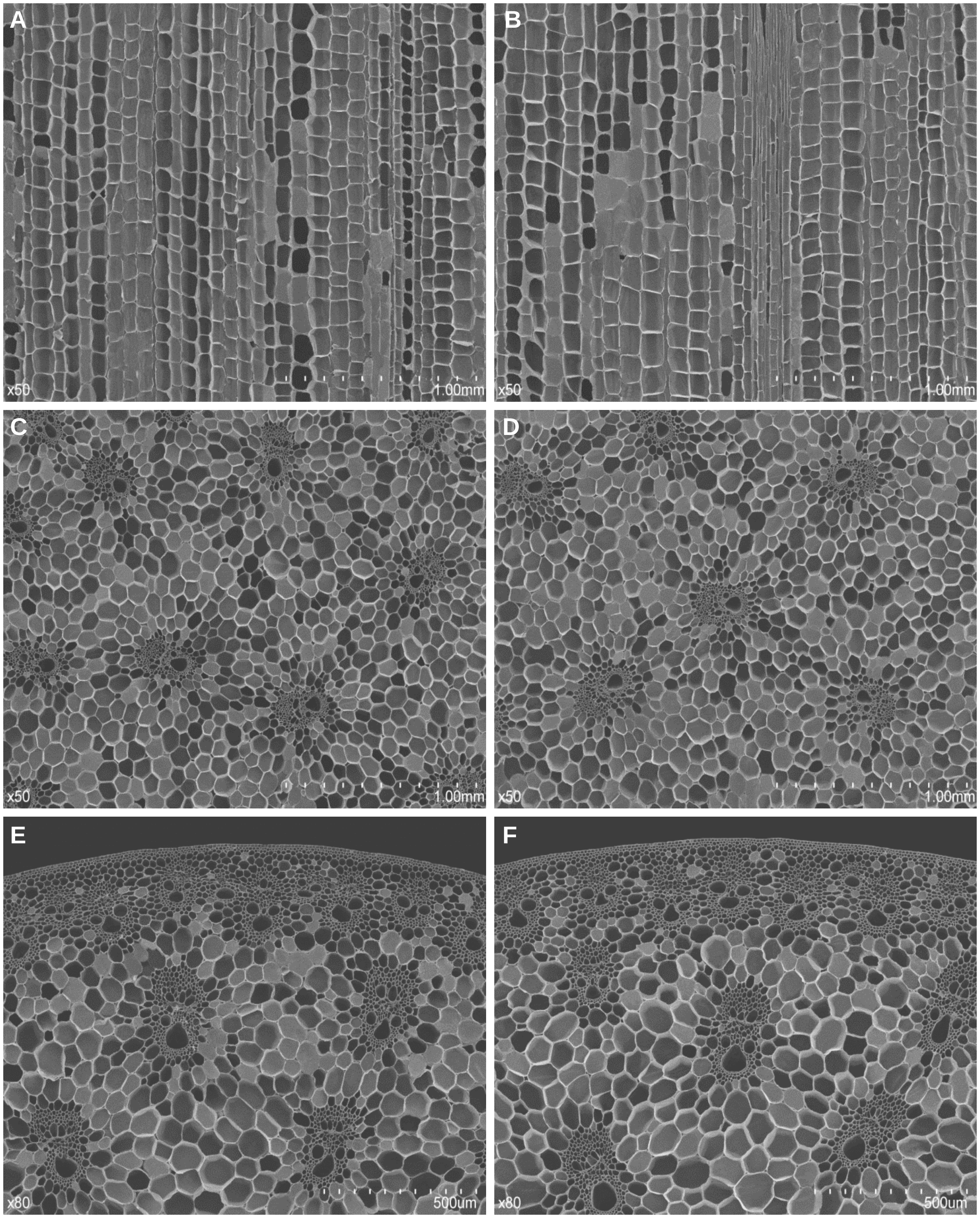

Figure S6. Scanning electron microscopy examination of the uppermost internode from RIL88$_{(qph1)}$ and RIL88$_{(QPH1)}$.

The uppermost internode of RIL88$_{(qph1)}$ and RIL88$_{(QPH1)}$ in adult stage were subjected to histological analysis. A and B, longitudinal sections of the parenchyma cells in RIL88$_{(qph1)}$ (left) and RIL88$_{(QPH1)}$ (right); C and D, transverse view of the parenchyma cells in RIL88$_{(qph1)}$ and RIL88$_{(QPH1)}$; E and F, transverse view of the epidermal region of RIL88$_{(qph1)}$ and RIL88$_{(QPH1)}$.

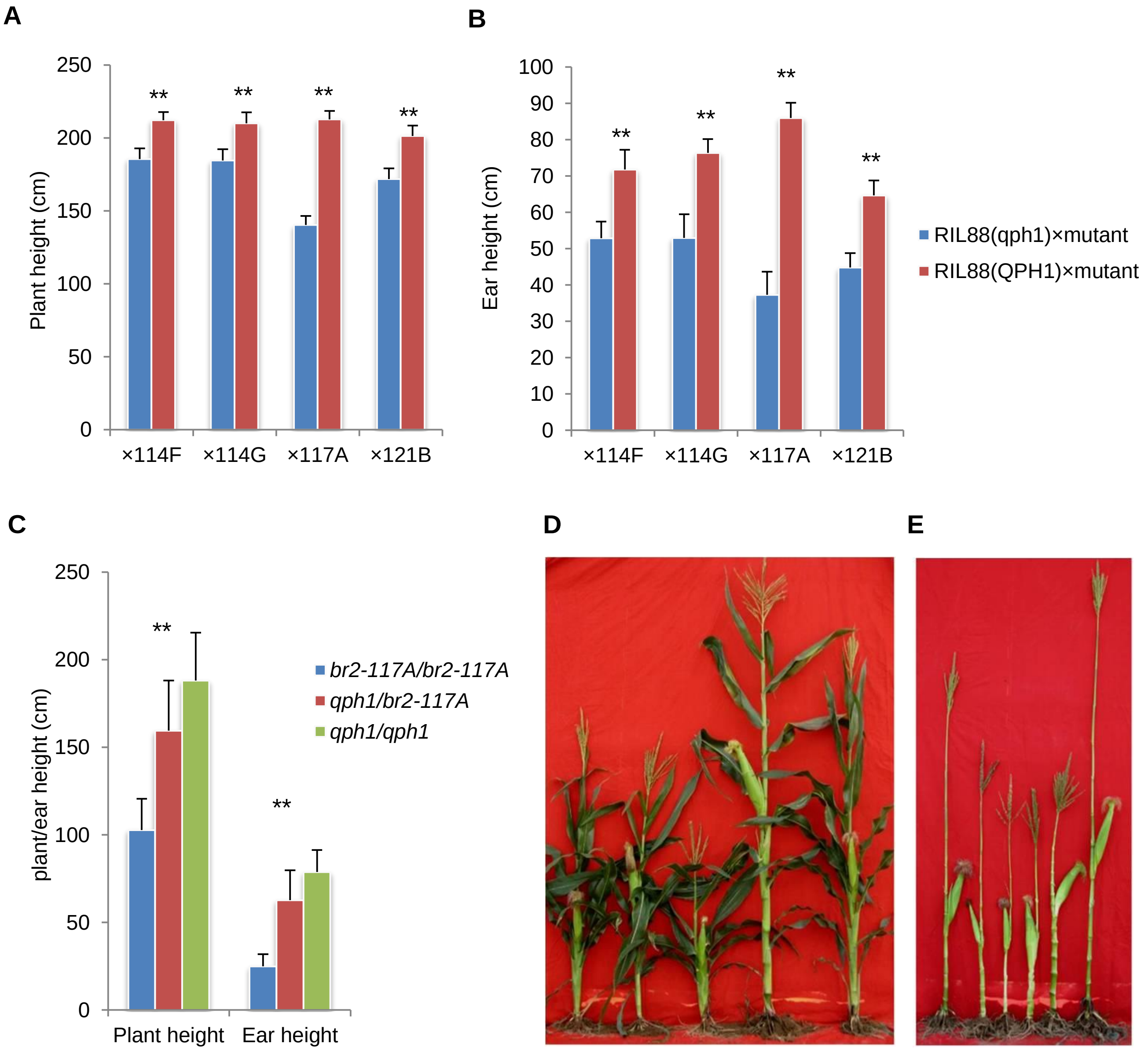

Figure S7. Allelism test of *qph1*.

A. Plant height comparison between hybrids derived by RIL88$_{(qph1)}$×*br2* mutants and RIL88$_{(QPH1)}$×*br2* mutants. B. Ear height comparison between hybrids derived by RIL88$_{(qph1)}$×*br2* mutants and RIL88$_{(QPH1)}$×*br2* mutants. C. Significance test of Plant height and ear height between *br2-117a/br2-117a, br2-117a/qph1 and qph1/qph1* individuals in the RIL88$_{(qph1)}$×117A F_2 population. D. RIL88$_{(qph1)}$, RIL88$_{(qph1)}$×117A, 117A, RIL88$_{(QPH1)}$×117A and RIL88$_{(QPH1)}$. E. Plant height and ear height performance of RIL88$_{(qph1)}$, RIL88$_{(QPH1)}$ and the four *br2* mutants (from left to right: RIL88$_{(qph1)}$, 114F, 114G, 117A, 121B and RIL88$_{(QPH1)}$). Error bars indicate S.D.

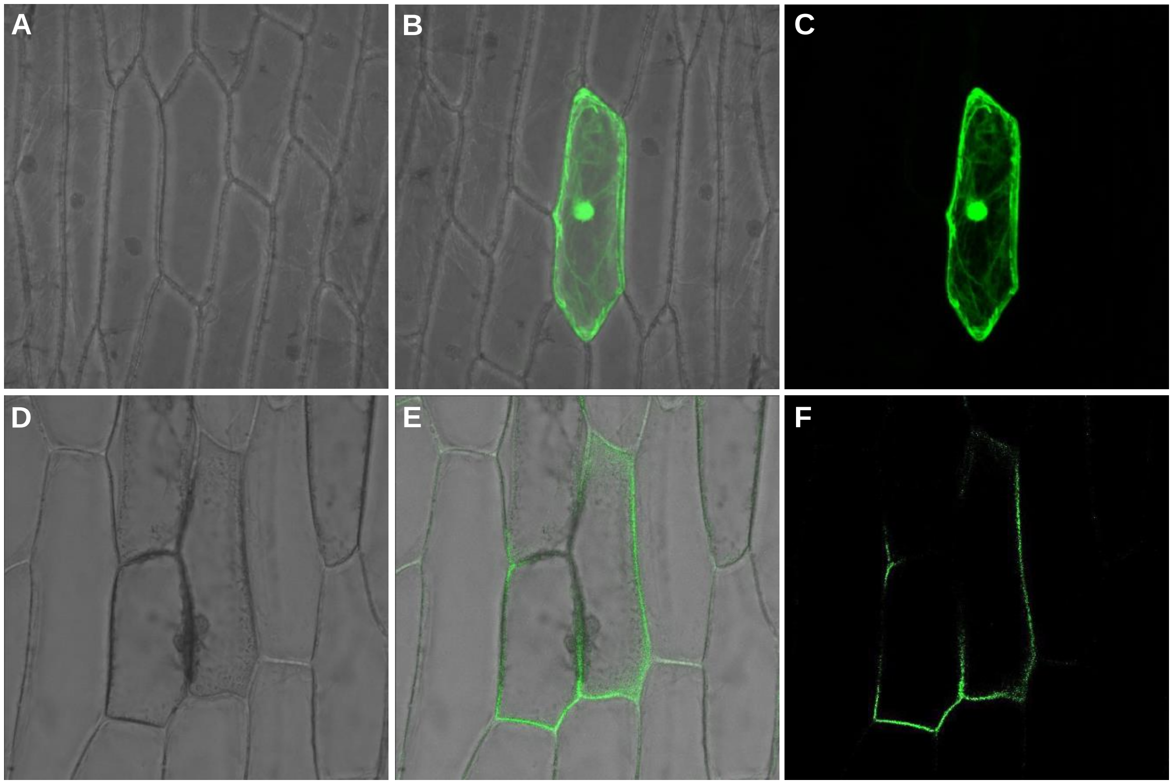

Figure S8. Sub-cellular localization of *QPH1*.

(A-C) Transient expression of GFP in onion epidermal cells in bright (A), merge (B) and dark (C) field. (D-F) Transient expression of *QPH1-GFP* in onion epidermal cells in bright (D), merge (E) and dark (F) field.

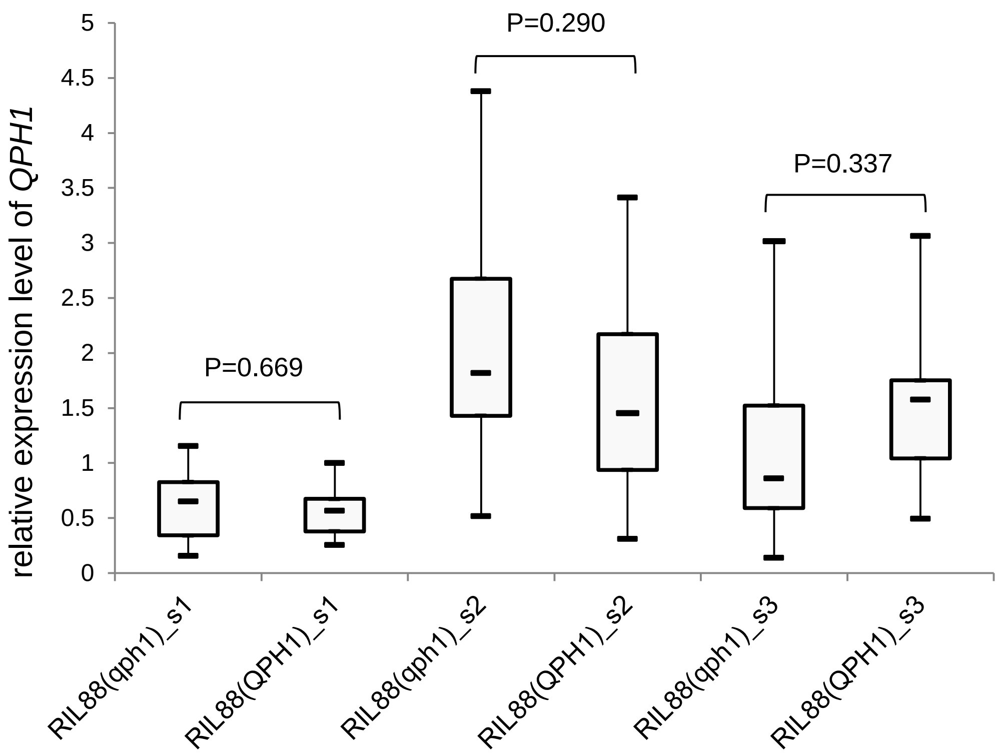

Figure S9. *qph1* expression in RIL88$_{(qph1)}$ and RIL88$_{(QPH1)}$ internodes from three developmental stages during elongation.

s1, s2 and s3 represent three different developmental stages of RIL88$_{(qph1)}$ and RIL88$_{(QPH1)}$ during elongation. In stage 1 (s1), which is at the beginning of elongation stage, plants have 8 expanded leaves and 13 visible leaves; in stage 2 (s2), which is in the middle of elongation, plants have 9 expanded leaves and 14 visible leaves; in stage 3 (s3), which is near the end of elongation, plants have 11 expanded leaves and 16 visible leaves. Relative expression level of *QPH1* (*qph1*) to the endogenous control zmβ-actin is shown.

A B

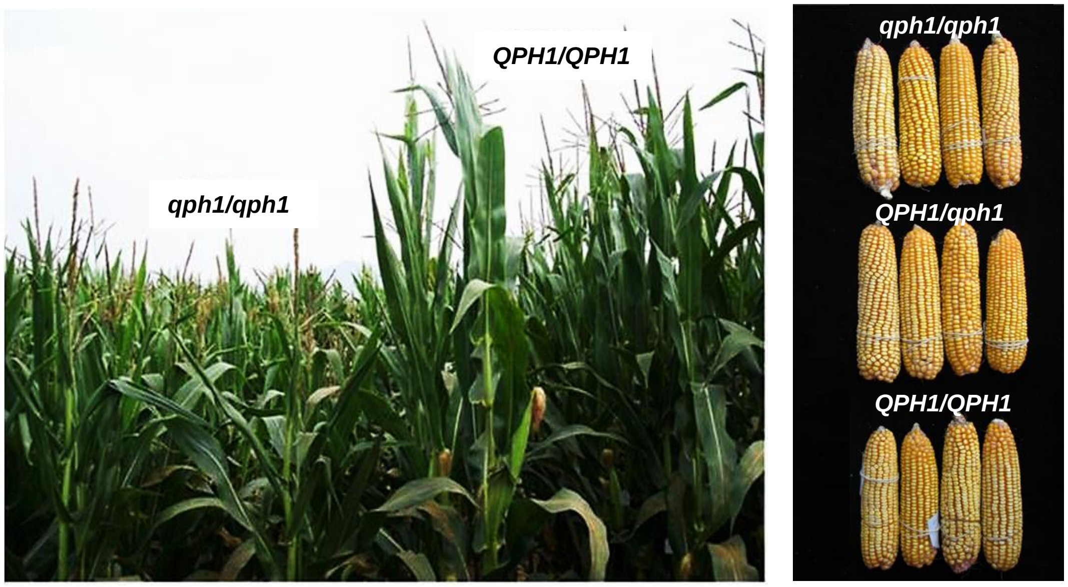

Figure S10. Plant height (A) and yield performance (B) comparison between individuals of different genotypes in B73×Ye107 F_2 population.

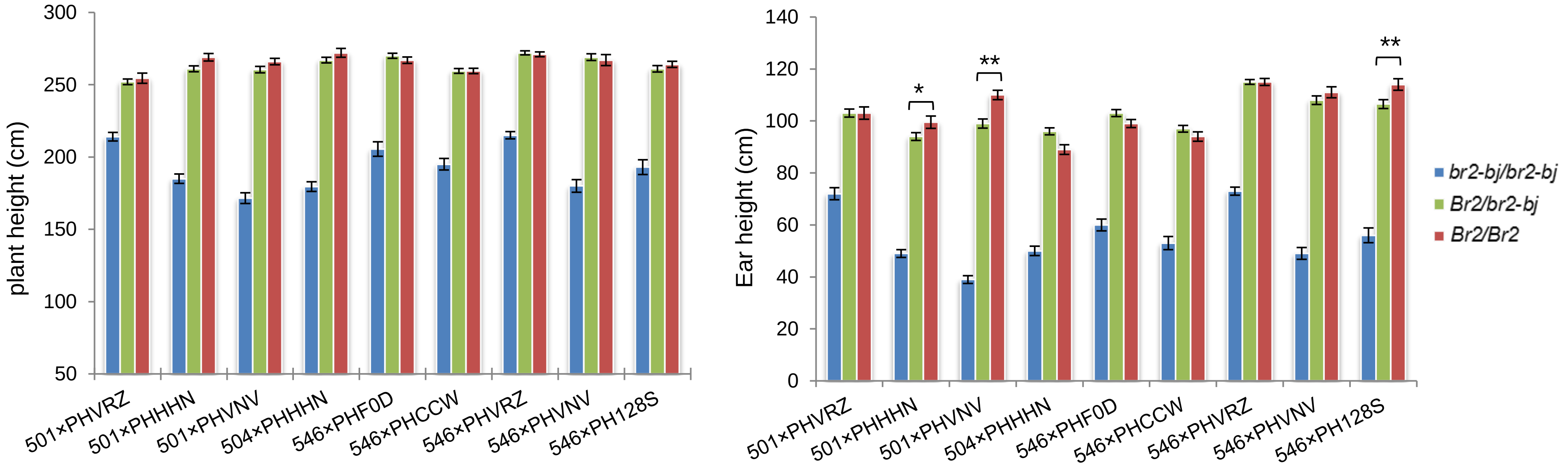

Figure S11. Plant height and ear height segregation in 9 F_2 populations of N546 conversion lines and normal inbred lines.

*Significant difference between *br2-bj/Br2* and *BR2/Br2* individuals (0.05 level)

**Highly significant different between *br2-bj/Br2* and *BR2/Br2* individuals (0.01 level)

Data is expressed as mean ± s.d.

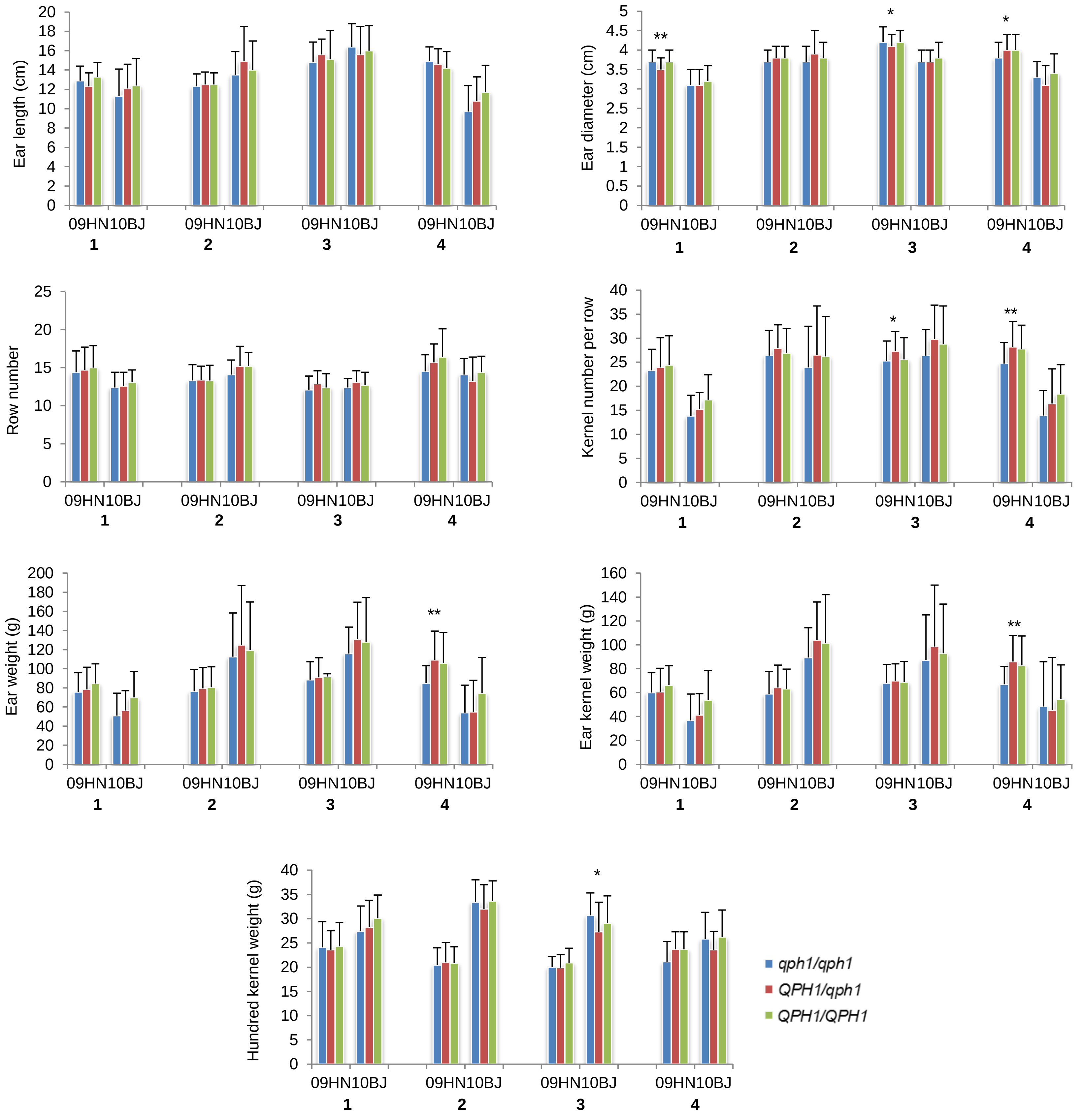

Figure S12. Yield component analysis of the four F_2 populations.

1, 2, 3, 4, represent 4F1×81162 , Ye107×Zheng32, Ye107×B73 and Zong3×Chuan48-2 F_2 populations

* Phentotype between individuals of different genotypes reached significant level (0.05)

** Phentotype between individuals of different genotypes reached significant level (0.01)
